# Cross-feeding by *Bifidobacterium breve* UCC2003 during co-cultivation with *Bifidobacterium bifidum* PRL2010 in a mucin-based medium

**DOI:** 10.1186/s12866-014-0282-7

**Published:** 2014-11-25

**Authors:** Muireann Egan, Mary O’Connell Motherway, Michelle Kilcoyne, Marian Kane, Lokesh Joshi, Marco Ventura, Douwe van Sinderen

**Affiliations:** School of Microbiology and Alimentary Pharmabiotic Centre, University College Cork, Cork, Ireland; Glycoscience Group, National Centre for Biomedical Engineering Science, National University of Ireland, Galway, Ireland; Microbiology, School of Natural Sciences, National University of Ireland, Galway, Ireland; Laboratory of Probiogenomics, Department of Life Sciences, University of Parma, Parma, Italy

**Keywords:** Bifidobacteria, Probiotic, Fucose, Sialic acid, Galactose

## Abstract

**Background:**

Bifidobacteria constitute a specific group of commensal bacteria that commonly inhabit the mammalian gastrointestinal tract. *Bifidobacterium breve* UCC2003 was previously shown to utilize a variety of plant/diet/host-derived carbohydrates, including cellodextrin, starch and galactan, as well as the mucin and HMO-derived monosaccharide, sialic acid. In the current study, we investigated the ability of this strain to utilize parts of a host-derived source of carbohydrate, namely the mucin glycoprotein, when grown in co-culture with the mucin-degrading *Bifidobacterium bifidum* PRL2010.

**Results:**

*B. breve* UCC2003 was shown to exhibit growth properties in a mucin-based medium, but only when grown in the presence of *B. bifidum* PRL2010, which is known to metabolize mucin. A combination of HPAEC-PAD and transcriptome analyses identified some of the possible monosaccharides and oligosaccharides which support this enhanced co-cultivation growth/viability phenotype.

**Conclusion:**

This study describes the potential existence of a gut commensal relationship between two bifidobacterial species. We demonstrate the *in vitro* ability of *B. breve* UCC2003 to cross-feed on sugars released by the mucin-degrading activity of *B. bifidum* PRL2010, thus advancing our knowledge on the metabolic adaptability which allows the former strain to colonize the (infant) gut by its extensive metabolic abilities to (co-)utilize available carbohydrate sources.

## Background

Bifidobacteria are Gram positive, anaerobic, Y-shaped bacteria that have been found in the gastrointestinal tract (GIT) of mammals, birds and insects and have also been isolated from the human oral cavity and sewage [1]. In recent years, bifidobacteria have attracted attention due to the purported health benefits associated with their presence in the gut. Such beneficial contributions include development and modulation of the immune system [2], provision of vitamins [3], and protection against pathogenic bacteria [4]. Bifidobacteria rapidly colonize the infant gut in the first days and weeks of life. In a recent study on the microbial diversity of the infant gut, it was found that members of the *Actinobacteria* phylum were dominant, with various bifidobacterial species present in high abundance, in particular *Bifidobacterium longum*, *Bifidobacterium bifidum, Bifidobacterium breve* and *Bifidobacterium catenulatum* [5]*.*

Survival and growth of bifidobacteria in the gastrointestinal tract requires them to employ an arsenal of enzymes to metabolize the complex carbohydrates prevalent in this environment [6]. *B. breve* UCC2003 has previously been shown to utilize various diet/plant-derived oligo- and poly-saccharides, including melezitose, raffinose, cellodextrins, GOS, starch and galactan [7-12]. Recently, *B. breve* UCC2003 was shown to utilize the mucin- and human milk oligosaccharide (HMO)-derived monosaccharide sialic acid [13], which is more consistent with this strain’s origin as a nursling stool isolate from a breast-fed infant, where it would be expected to metabolize HMOs and/or the structurally similar oligosaccharides found in mucin glycoproteins. Along with dietary components, host-derived oligosaccharides are believed to form part of the nutrient resource for certain intestinal bacteria.

The original investigations on the (bio)chemical composition of human colonic mucin described twenty-one discrete oligosaccharide structures [14]. Since then, it has been estimated that carbohydrate constitutes approximately 80% of the total mucin mass, and that MUC2, the prominent secretory mucin in the colon, may contain more than 100 different *O-*linked glycans [15,16]. Glycosylation of the peptide backbone can be *N-*linked to an asparagine residue or *O-*linked to serine or threonine residues via *N*-acetylgalactosamine (GalNAc). Subsequent elongation of this structure results in a number of distinct core structures. While eight mucin type core structures are known, only four regularly occur in human mucins. Core 1 is formed by the addition of galactose (Gal) in a β-(1,3) linkage to GalNAc to produce galacto-*N*-biose (GNB, also known as the T antigen). Core 3, the most common core structure in the human colon, is formed by the addition of *N*-acetylglucosamine (GlcNAc) in a β-(1,3) linkage to GalNAc. Core 2 and core 4 structures are formed by the addition of GlcNAc in a β-(1,6) linkage to core 1 and core 3, respectively. Each of the core structures can be further elongated by the addition of Gal, GalNAc and GlcNAc. The oligosaccharide chains can also be substituted with sialic acid, fucose (Fuc) or sulfate residues in terminal or branched positions [14,15,17,18].

Due to the high complexity and variability of the mucin oligosaccharide chains, only a small proportion of the culturable intestinal microbiota is believed to encode enzymes required for (partial) degradation of mucin into free sugars. These include members of the *Bifidobacterium, Bacteroides* and *Ruminococcus* genera, as well as a more recently characterized bacterium isolated from human faeces, *Akkermansia muciniphila* [19,20]. The degradation of mucin occurs sequentially with the removal of component monosaccharides, rather than the removal of the entire polysaccharide structure, thus requiring a multitude of enzymes with various glycosidic specificities [21].

The first observation of mucin-degrading bifidobacteria was described by Hoskins *et al.*, who described the isolation of two bifidobacterial strains that constitutively expressed extracellular enzymes capable of degrading oligosaccharide side chains of gut mucins [22]. Cell surface-anchored glycosyl hydrolases were later characterized, including two α-L-fucosidases, AfcA and AfcB, from *B. bifidum* JCM1254 [23,24], and an endo-α-*N*-acetylgalactosaminidase, EngBF, from *B. longum* JCM1217, which hydrolyzes the linkage between GalNAc of the core 1 disaccharide and the serine or threonine residue of the proteinaceous backbone [25].

As mentioned previously, *B. bifidum* is one of the most abundant species in the infant intestine [5]. A study in 2010 revealed that some 60% of the glycosyl hydrolases identified on the genome of *B. bifidum* PRL2010 are linked to the degradation of mucin [26,27]. The identified glycosyl hydrolases include two putative exo-α-sialidases, two putative α-L-fucosidases and a cell wall-anchored endo-α-*N*-acetylgalactosaminidase [26]. Other mucin-degrading enzymes on the genome of *B. bifidum* PRL2010 include four *N-*acetyl-β-hexosaminidases and four β-galactosidases [26,27]. Comparative genome hybridization analysis revealed that most of these genes encoding the above mentioned enzymes are conserved within the examined members of the *B. bifidum* species [26,27].

It has been suggested that the degradation of mucin by a small number of extracellular glycosidase-producing bacteria may provide nutritional support to other enteric bacteria [22]. A similar concept was more recently suggested in relation to the metabolism of HMO [28] and it has been shown that a number of HMO-derived degradation products remain in the media during vegetative growth of *B. bifidum* [29]. We recently demonstrated that *B. breve* UCC2003 can indeed cross-feed on sialic acid derived from the metabolism of 3′ sialyllactose, an abundant HMO, by *B. bifidum* PRL2010 [13]. The aim of the current study was to establish if *B. breve* UCC2003 is able to cross-feed on the oligosaccharides released by *B. bifidum* PRL2010 extracellular activity on mucin, and secondly, to investigate which, if any, particular components of mucin *B. breve* UCC2003 utilizes under such circumstances.

## Methods

### Bacterial strains, plasmids, media and culture conditions

Bacterial strains and plasmids used in this study are listed in Table 1. *B. breve* UCC2003 and its derivatives were routinely cultured in Reinforced Clostridial Medium (RCM; Oxoid Ltd, Basingstoke, Hampshire, United Kingdom). *B. bifidum* PRL2010 was routinely cultured in modified deMan Rogosa Sharpe (mMRS) medium made from first principles (but excluding a carbohydrate source) [30], and supplemented with 0.05% (wt/vol) L-cysteine HCl and 1% (wt/vol) lactose (unless otherwise stated). All carbohydrates used in this study were purchased from Sigma Aldrich and were of the highest purity available. To prepare mucin-containing media, a 0.8% (wt/vol) concentration of mucin from porcine stomach (Type III) was prepared in water and autoclaved at 115°C for 10 minutes, in order to optimize mucin dissolution, yet minimizing glycosidic hydrolysis. This was added to an equal volume of twice-concentrated mMRS, resulting in a final concentration of 0.4% mucin in mMRS. Bifidobacterial cultures were incubated under anaerobic conditions in a modular atmosphere-controlled system (Davidson and Hardy, Belfast, Ireland) at 37°C. *Escherichia coli* was cultured in Luria Bertani (LB) broth at 37°C with agitation [31]. Where appropriate, growth media contained tetracycline (Tet; 10 μg ml^−1^), chloramphenicol (Cm; 5 μg ml^−1^ for *E. coli,* 2.5 μg ml^−1^ for *B. bifidum*), erythromycin (Em; 100 μg ml^−1^) or kanamycin (Kan; 50 μg ml^−1^).

**Table 1:** **Bacterial strains and plasmids used in this study**

**Strains and plasmids**	**Relevant features**	**Reference or source**
**Strains**		
***Escherichia coli*** **strains**		
***E.coli*** **EC101**	Cloning host; *repA* ^+^ *kmr*	[37]
***E.coli*** **EC101-pNZ-M.Bbrll + Bbr11**	EC101 harboring a pNZ8048 derivative containing bbrllM and bbrlllM	[39]
***Bifidobacterium*** **sp. strains**		
***B. breve*** **UCC2003**	Isolate from a nursling stool	[36]
***B. breve*** **UCC2003** ***-*** **pAM5**	*B. breve* UCC2003 harboring pAM5	This study
***B. breve*** **UCC2003-nanA**	pORI19-Tet-nanA (Bbr_0168) insertion mutant of *B. breve* UCC2003	[13]
***B. breve*** **UCC2003-fucP**	pORI19-Tet-fucP (Bbr_1742) insertion mutant of *B. breve* UCC2003	This study
***B. breve*** **UCC2003-lnbP**	pORI19-Tet-lnbP (Bbr_1587) insertion mutant of *B. breve* UCC2003	This study
***B. breve*** **UCC2003-lacZ7**	pORI19-Tet-lacZ7 (Bbr_1833) insertion mutant of *B. breve* UCC2003	This study
***B. breve*** **UCC2003-galT**	Tet^r^ transposon mutant of *B. breve* UCC2003	[41]
***B. bifidum*** **PRL2010**	Isolate from infant faeces	[26]
***B. bifidum*** **PRL2010-pPKCM7**	*B. bifidum* PRL2010 harboring pPKCM7	This study
**Plasmids**		
**pAM5**	pBC1-puC19-Tc^r^	[38]
**pPKCM7**	pblueCm harboring rep pCIBA089	[40]
**pORI19**	Em^r^, repA^−^, ori^+^, cloning vector	[37]
**pORI19-tetW-fucP**	Internal 400 bp fragment of *fucP* and *tetW* cloned in pORI19	This study
**pORI19-tetW-lnbP**	Internal 479 bp fragment of *lnbP* and *tetW* cloned in pORI19	This study
**pORI19-tetW-lacZ7**	Internal 568 bp fragment of *lacZ7* and *tetW* cloned in pORI19	This study

### Nucleotide sequence analysis

Sequence data were obtained from the Artemis-mediated genome annotations of *B. breve* UCC2003 [32,33]. Database searches were performed using non-redundant sequences available at the National Centre for Biotechnology Information internet site (http://www.ncbi.nlm.nih.gov) using BLAST [34]. Sequence analysis was performed using the Seqbuilder and Seqman programs of the DNASTAR software package (DNASTAR, Madison, WI, USA).

### DNA manipulations

Chromosomal DNA was isolated from *B. breve* UCC2003 as previously described [35]. Plasmid DNA was isolated from *E. coli*, *B. breve* and *B. bifidum* using the Roche High Pure plasmid isolation kit (Roche Diagnostics, Basel, Switzerland). An initial lysis step was performed using 30 mg ml^−1^ of lysozyme for 30 min at 37°C prior to plasmid isolation from bifidobacteria. Single stranded oligonucleotide primers used in this study were synthesized by Eurofins (Ebersberg, Germany) (Table 2). PCRs were performed using Taq PCR master mix (Qiagen GmbH, Hilden, Germany). PCR products were purified using the Roche High Pure PCR purification kit (Roche Diagnostics). Electroporation of plasmid DNA into *E. coli* or bifidobacteria was performed as described previously [31,36].

**Table 2:** **Oligonucleotide primers used in this study**

**Purpose**	**Primer**	**Sequence**
**Cloning of 400 bp fragment of** ***fucP*** **(Bbr_1742) in pORI19**	FucPF	TAGCAT*AAGCTT*GGCGAATCGTTCGTATCA
	FucPR	GATATC*TCTAGA*GCGCCCCAGTGCTTGAGC
**Cloning of 479 bp fragment of** ***lnbP*** **(Bbr_1587) in pORI19**	LnbPF	TAGCAT*AAGCTT*CACACAGGTATTGGGAGGTTG
	LnbPR	CTAGTC*TCTAGA*GTTGTAGGCGCCACCATCC
**Cloning of 568 bp fragment of** ***lacZ7*** **(Bbr_1833) in pORI19**	LacZ7F	TAGCAT*AAGCTT*CCAGGCCAAGAACTCCAGTG
	LacZ7R	CATGAT*TCTAGA*CAGCTTGGGCAGGTTGAACG
**Amplification of** ***tetW***	TetWF	TCAGCT*GTCGAC*ATGCTCATGTACGGTAAG
	TetWR	GCGACG*GTCGAC*CATTACCTTCTGAAACATA
**Confirmation of site-specific homologous recombination**	FucPconfirm	TGTTCGCCATGTTCGTTATC
	LnbPconfirm	GATCACTCTGCATATGGACG
	LacZ7confirm	GTACCGACATCGACGCGTTC

Restriction sites incorporated into oligonucleotide primer sequences are indicated in italics.

### Construction of *B. breve* UCC2003 insertion mutants

An internal fragment of Bbr_1742, designated here as *fucP* (400 base pairs (bp), representing codon numbers 137 through to 270 out of the 421 codons of *fucP*), Bbr_1587, designated here as *lnbP* (479 bp, representing codon numbers 108 through to 267 of the 751 codons of *lnbP*) and Bbr_1833, designated here as *lacZ7* (568 bp, representing codon numbers 123 through 312 of the 699 codons of *lacZ7*), were amplified by PCR using *B. breve* UCC2003 chromosomal DNA as a template and primer pairs FucPF and FucPR, LnbPF and LnbPR and LacZ7F and LacZ7R, respectively. The generated amplicons were digested with HindIII and XbaI, and ligated to the similarly digested pORI19, an ORI^+^, RepA^−^ integration plasmid [37]. The ligation mixtures were introduced into *E. coli* EC101 by electroporation and transformants were selected on LB agar containing Em and supplemented with 40 mg ml^−1^ X-Gal (5-bromo-4-chloro-3-indolyl-β-D-galactopyranoside) and 1 mM IPTG (isopropyl-β-D-galactopyranoside). The plasmid content of a number of Em^r^ transformants was screened by restriction analysis and the integrity of positively identified clones was verified by sequencing. This was followed by subcloning of the Tet antibiotic resistance cassette, *tetW* from pAM5 [38] as a SacI fragment into the unique SacI site present on each pORI19 derivative. The orientation of the *tetW* fragment in each of the resulting plasmids, pORI19-tetW-fucP, pORI19-tetW-lnbP and pORI 19-tetW-lacZ7, was verified by restriction analysis, followed by the introduction of the plasmid by electroporation into the *E. coli* EC101 pNZ-MBbrI-MBbrII strain to methylate the plasmid constructs prior to introduction into *B. breve* UCC2003 [39]. Methylation of the plasmids was confirmed by their observed resistance to PstI digestion [39]. The methylated plasmids, pORI19-tetW-fucP, pORI19-tetW-lnbP and pORI19-tetW-lacZ7, were introduced into *B. breve* UCC2003 by electroporation and transformants were selected on Reinforced Clostridial Agar (RCA) supplemented with Tet. Site-specific recombination in potential Tet-resistant mutant isolates was confirmed by colony PCR using primer combinations TetWF and TetWR to confirm *tetW* gene integration, and the primers FucPconfirm, LnbPconfirm and LacZ7confirm (positioned upstream of the selected internal fragment of *fucP, lnbP* and *lacZ7*, respectively) and TetWF to confirm integration at the correct chromosomal location.

### Evaluation of *B. breve* UCC2003 growth on mucin

Growth of *B. breve* UCC2003 using mucin as the sole carbon source was determined both independently and in co-culture with *B. bifidum* PRL2010. Plasmid-containing derivatives of the wild type strains were used, namely *B. breve* UCC2003-pAM5, which contains the pAM5 plasmid and is therefore Tet resistant [38] and *B. bifidum* PRL2010-pPKCM7, which contains the pPKCM7 plasmid and is Cm resistant [40]. Use of plasmid-containing derivatives allowed for selection and enumeration of either *B. breve* UCC2003 or *B. bifidum* PRL2010 colonies on RCA containing the appropriate antibiotic. All *B. breve* UCC2003 mutant strains were also Tet resistant. A 0.001% inoculum of a stationary phase culture of *B. breve* UCC2003 strains (see Table 1; *B. breve* UCC2003-pAM5, or the mutant strains, *B. breve* UCC2003-nanA [13], *B. breve* UCC2003-fucP, *B. breve* UCC2003-lnbP, *B. breve* UCC2003-lacZ7 or *B. breve* UCC2003-galT [41]) and/or a 0.01% inoculum of *B. bifidum* PRL2010-pPKCM7, were added to mMRS medium supplemented with 0.05% (wt/vol) L-cysteine HCl and 0.4% (wt/vol) mucin (see above). Growth of the cultures was measured over 72 h, with samples taken every 6 or 12 h. All samples collected were serially 10-fold diluted in sterile Ringers solution and plated onto RCA supplemented with 1% (wt/vol) lactose and the appropriate antibiotic. Viable counts were determined by counting colonies on agar plates using dilutions that yielded between 30 and 300 colony forming units (CFU).

### Transcriptome analysis using *B. breve* UCC2003-based microarrays

*B. breve* UCC2003-pAM5 was grown in mMRS medium supplemented with 0.05% (wt/vol) L-cysteine HCl and 0.4% ribose to an OD_600nm_ of 0.5 and then harvested by centrifugation at 9,000 × *g* for 2 min at room temperature. *B. breve* UCC2003-pAM5 grown in mucin in co-culture with *B. bifidum* PRL2010-pPKCM7 (see above) was similarly harvested after 30 h of growth. DNA microarrays containing oligonucleotide primers representing each of the 1864 annotated genes on the genome of *B. breve* UCC2003 were designed by and obtained from Agilent Technologies (Palo Alto, CA, USA). Methods for cell disruption, RNA isolation, RNA quality control, complementary DNA (cDNA) synthesis and labelling were performed as described previously [42]. Labelled cDNA was hybridized using the Agilent Gene Expression hybridization kit (part number 5188–5242) as described in the Agilent Two-Color Microarray-Based Gene Expression Analysis (v4.0) manual (publication number G4140-90050). Following hybridization, microarrays were washed as described in the manual and scanned using Agilent’s DNA microarray scanner G2565A. The scanning results were converted to data files with Agilent’s Feature Extraction software (version 9.5). DNA-microarray data were processed as previously described [43-45]. Differential expression tests were performed with the Cyber-T implementation of a variant of the *t*-test [46].

### Analysis of the monosaccharide composition of fermented and non-fermented mucin

Identification and quantification of the free monosaccharides in non-fermented mMRS supplemented with 0.4% mucin and the media following 30 h fermentation by *B. bifidum* PRL2010-pPKCM7 was performed according to Dionex technical note 40 (http://www.dionex.com/en-us/webdocs/5052-TN40-IC-Glycoprotein-Monosaccharide-23May2012-LPN1632-01.pdf) and as previously described [47], using high performance anion exchange chromatography with pulsed amperometric detection (HPAEC-PAD). In brief, media was pelleted out, sterilized through a 0.2 μm filter and then lyophilized to dryness. The lyophilized powder was dissolved at 1 mg ml^−1^ in purified water. Dilutions were injected on to a Dionex ICS 3000 system (Dionex, Sunnyvale, CA) equipped with a CarboPac PA20 analytical column (150 mm × 3 mm) with an Amino-trap column (30 mm × 3 mm) and separated as previously described [48]. Resulting peaks were identified and quantified by comparison to a standard curve with a mixture containing the common mammalian residues Fuc, glucosamine (GlcN), galactosamine (GalN), Gal, glucose (Glc) and mannose (Man). Samples were injected three times and the average value is reported for the concentration. Samples were then spiked with a known concentration of Fuc and Gal standards and re-injected to confirm the identification of these residues in the sample.

To further confirm the identity of Fuc in spent medium, samples were labelled with 2-aminobenzamide (2-AB) according to the published method [49]. The 2-AB labelled samples were cleaned on Glycoclean S cartridges (Prozyme) according to manufacturer’s instructions and vacuum centrifuged dry. Samples were analyzed by reverse phase-high performance liquid chromatography (RP-HPLC) injected onto a Waters Alliance 2695 instrument and separated on a Phenomenex Luna 3u C18(2) (150 mm × 4.6 mm) column using previously described conditions [50].

During co-culture experiments samples of cell free supernatants were taken every 6 or 12 h to qualitatively analyze the carbohydrate composition of the media by HPAEC-PAD. Samples (25 μl aliquots) were separated on a CarboPac PA1 analytical-exchange column (250 mm × 4 mm) with a CarboPac PA1 guard column (50 mm × 4 mm). Elution was performed at a constant flow-rate of 1.0 ml min^−1^ at 30°C using the following eluents for the analysis (A) 200 mM NaOH, (B) 100 mM NaOH, 550 mM sodium acetate, and (C) purified water. The following linear gradient of sodium acetate was used with 100 mM NaOH: from 0 to 50 min, 0 mM; from 50 to 51 min, 16 mM; from 51 to 56 min, 100 mM; from 56 to 61 min, 0 mM. Eluate was monitored with a Dionex ED40 detector in the PAD mode. The chromatogram of non-fermented mucin was used to evaluate mucin utilization by *B. bifidum* PRL2010-pPKCM7 and *B. breve* UCC2003-pAM5, and the Chromeleon software v. 6.70 (Dionex Corporation) was used for the integration and evaluation of the chromatograms obtained.

### Microarray data accession number

The microarray data obtained in this study have been deposited in NCBI’s Gene Expression Omnibus database and are accessible through GEO series accession number GSE59013.

### Ethics statement

No animal or human subjects were involved in this study.

## Results

### Growth of *B. breve* UCC2003 on mucin

**Figure 1:**
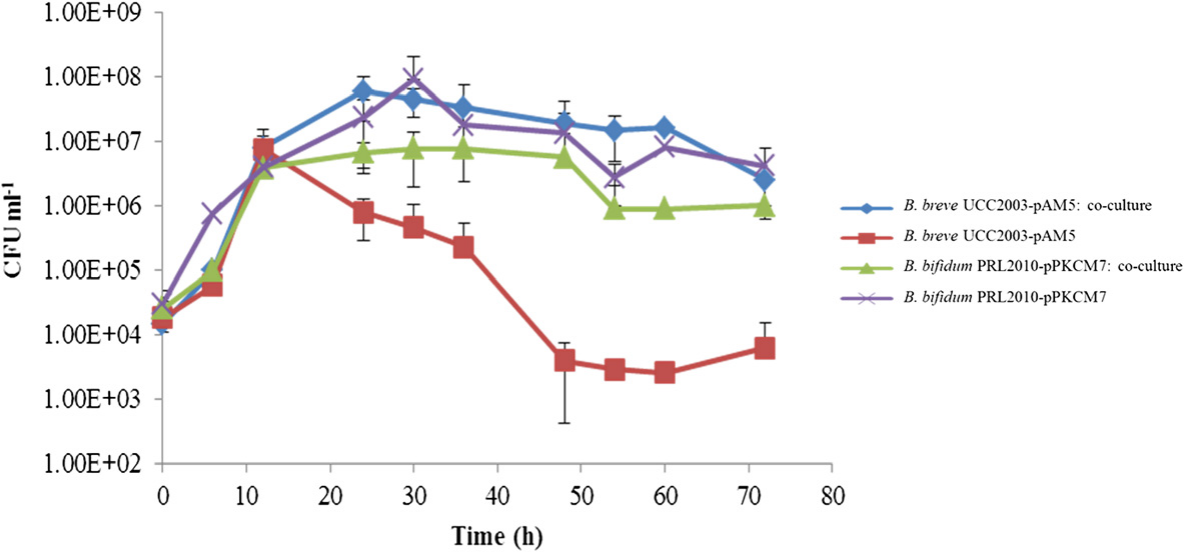
**Individual and co-culture growth profiles of** ***B. breve*** **UCC2003-pAM5 and** ***B. bifidum*** **PRL2010-pPKCM7 in mucin.** All growth experiments were performed in mMRS supplemented with 0.4% mucin from porcine stomach for 72 h. The results presented are the mean values of duplicate experiments. Error bars represent the standard deviation.

Growth of *B. breve* UCC2003-pAM5 and *B. bifidum* PRL2010-pPKCM7 in mMRS supplemented with 0.4% (wt/vol) mucin, independently or in co-culture, was measured by viable plate counts over 72 h. Monitoring of growth in co-culture required the use of plasmid-containing derivatives of the wild type strains. The plasmids used, pAM5 for *B. breve* UCC2003 or pPKCM7 for *B. bifidum* PRL2010, conferred Tet or Cm resistance to the respective strains, thus allowing selection and enumeration of each strain by viable plate count on RCA containing the corresponding antibiotic. Both *B. breve* UCC2003-pAM5 and *B. bifidum* PRL2010-pPKCM7 were also cultured in mMRS supplemented with 0.5% lactose, a substrate on which both strains achieve a high level of growth [51,52], and it was found that presence of the plasmid did not impair growth of the strain on this substrate (results not shown). A low inoculum of 0.001% and/or 0.01% of *B. breve* UCC2003-pAM5 and *B. bifidum* PRL2010-pPKCM7, respectively, was used to allow the strains to undergo multiple generations of growth. In the absence of an added carbohydrate, no growth was observed for either *B. breve* UCC2003-pAM5 or *B. bifidum* PRL2010-pPKCM7 in mMRS medium (results not shown). As expected, the positive control, *B. bifidum* PRL2010-pPKCM7, was capable of growth in mucin-containing mMRS medium, from a low inoculum of 10^4^ CFU ml^−1^ it attained cell numbers of approximately 10^8^ CFU ml^−1^ following 36 h of incubation [26] (Figure 1). However, a notably different growth profile was observed for *B. breve* UCC2003-pAM5 on this medium. During the first 12 h of incubation in this medium, *B. breve* UCC2003-pAM5 viable cell numbers increased from 10^4^ CFU ml^−1^ to almost 10^7^ CFU ml^−1^, followed by a decline in viable cells to below 10^4^ CFU ml^−1^ (Figure 1). HPAEC-PAD analysis (see below) revealed the presence of contaminating monosaccharides, namely Glc and Gal, in the non-fermented mucin preparation. It is unknown whether these carbohydrates were released from partial degradation of the mucin during autoclaving, despite efforts made to reduce such degradation (see above), or were present as contaminating monosaccharides in the mucin preparation. Given that *B. breve* UCC2003 can utilize both of these contaminants as a sole carbon source [51], it is hypothesized that they are responsible for the observed growth during the first 12 h following inoculation. Once these carbon sources were utilized, and as *B. breve* UCC2003-pAM5 apparently does not have the required enzymes to degrade intact mucin, a substantial drop in viable count was observed. Interestingly, when *B. bifidum* PRL2010-pPKCM7 was included as a potential carbohydrate-releasing bacterium in co-culture with *B. breve* UCC2003-pAM5, an improvement in growth and survival abilities of *B. breve* UCC2003-pAM5 was observed. In co-culture, *B. breve* UCC2003-pAM5 reached cell numbers of almost 10^8^ CFU ml^−1^ after 24 h and maintained a viable count of over 10^6^ CFU ml^−1^ after 72 h (Figure 1). This suggests that *B. breve* UCC2003-pAM5 is cross-feeding on (some of) the sugars released following mucin breakdown by *B. bifidum* PRL2010-pPKCM7. The cell numbers of *B. bifidum* PRL2010-pPKCM7 were moderately lower in co-culture as compared to when the strain was grown independently in this medium and this was attributed to the presence of another acid-producing strain in the culture. However, the growth profile of *B. bifidum* PRL2010-pPKCM7 in co-culture followed a similar trend to that of the strain growing individually, suggesting that the two strains do not compete for limiting amounts of mucin-derived carbohydrates.

### Gene expression analysis using *B. breve* UCC2003 DNA microarrays

In order to investigate how the transcriptome of *B. breve* UCC2003-pAM5 is affected by growth in co-culture with *B. bifidum* PRL2010-pPKCM7 in mucin-containing mMRS, global gene expression was determined by microarray analysis during growth of the strain in co-culture and compared with gene expression when grown on ribose as the sole carbon source. Ribose was considered an appropriate carbohydrate for comparative transcriptome analysis as the genes involved in ribose metabolism are unique to this sugar [53], thus making it an ideal substrate for gene expression analysis on other carbohydrates. It has previously been used as a reference condition in several transcriptome studies in *B. breve* UCC2003 [7,9,10,13,54]. Furthermore, as a pentose sugar, ribose is suitable for gene expression analysis in mucin, given that the monosaccharide components of mucin are all hexose sugars (Glc, Gal, GalNAc, GlcNAc or Fuc), except sialic acid which is a nine-carbon monosaccharide. In the current study, gene expression was determined following 30 h growth in co-culture. This was to ensure that any observed changes in the transcriptome of *B. breve* UCC2003-pAM5 could be attributed solely to growth in co-culture with *B. bifidum* PRL2010-pPKCM7, as opposed to growth on free monosaccharides in the mucin preparation which appeared to be responsible for the initial 12 h of growth of *B. breve* UCC2003-pAM5 in mucin (see above). Analysis of DNA microarray data showed that a number of stress-related genes were up-regulated, which was attributed to the cells being in co-culture with another bacterium or to the possibility that the cells had entered the stationary phase. Nonetheless, the majority of genes that were shown to be significantly up-regulated (fold change >2.5, *P* < 0.001) in co-culture are predicted to be involved in the transport and metabolism of carbohydrates. One such cluster, Bbr_1740-1742, is believed to be involved in Fuc metabolism and includes genes predicted to encode a dihydrodipicolinate synthase (Bbr_1740), an enzyme which was previously shown to be involved in Fuc metabolism in *Campylobacter jejuni* [55]*,* a hypothetical protein (Bbr_1741) and a Fuc permease (*fucP,* Bbr_1742). Transcription of a predicted β-galactosidase-encoding gene (Bbr_1833, designated *lacZ7*) was also significantly increased, this enzyme is possibly involved in the removal of Gal from mucin oligosaccharides, suggesting that *B. breve* UCC2003 may utilize one or more Gal-containing fractions of mucin. The Bbr_1585-1590 cluster was also up-regulated during growth in co-culture. Bbr_1585 encodes a predicted UDP-Glc-4-epimerase, designated *galE.* Previously, UDP-Glc-4-epimerase enzymes have been shown to be involved in the Leloir pathway for Gal metabolism [56], as well as the GNB/LNB pathway for the metabolism of (ga)lacto-*N-*biose (GNB/LNB) derived from HMO or mucin [57,58]. Bbr_1586 encodes a predicted *N-*acetylhexosamine-1-kinase, designated *nahK*. In *B. longum* JCM 1217, an *N-*acetylhexosamine-1-kinase, which shares 90% identity to NahK, was shown to have a role in the previously mentioned GNB/LNB pathway [57]. Bbr_1587 encodes a predicted LNB phosphorylase, designated *lnbP*, an enzyme which is also involved in the GNB/LNB pathway [57-59]. The LnbP protein from *B. breve* UCC2003 shares 90% identity with the previously characterized LnbP protein from *B. bifidum* JCM 1254 [58]. Finally, as regards to this upregulated cluster, Bbr_1588-1590 encode a predicted ABC transport system, including two predicted permease proteins (represented by Bbr_1588 and Bbr_1589), and a solute binding protein (Bbr_1590), which shares 98% identity with the GNB/LNB-specific binding protein of *B. longum* JCM1217 [60]. Bbr_1884 (designated *galT2*), encoding a predicted Gal-1-phosphate uridyltransferase, another enzyme required for the Leloir pathway and the GNB/LNB pathway [56-58], was also up-regulated when *B. breve* UCC2003 was grown in mucin in co-culture with *B. bifidum* PRL2010. Finally, two gene clusters previously shown to be responsible for the transport and metabolism of sialic acid were significantly up-regulated under these growth conditions (Bbr_0160-0171 and Bbr_1247-1248) [13] (Table 3).

**Table 3:** **Effect of mucin (in co-culture with** ***B. bifidum*** **PRL2010) on the transcriptome of** ***B. breve*** **UCC2003**

**Gene No.**	**Predicted Function**	**Level of upregulation (** ***P*** **< 0.001)**
**Bbr_0161_** ***nanK***	Conserved hypothetical protein in ROK family	4.22
**Bbr_0162_** ***nanE***	*N*-acetylmannosamine-6-phosphate 2-epimerase	5.46
**Bbr_0164_** ***nanB***	ABC transport system, solute binding protein	14.08
**Bbr_0165_** ***nanC***	ABC transport system, permease protein	11.82
**Bbr_0166_** ***nanD***	ABC transport system, ATP-binding protein	18.92
**Bbr_0167_** ***nanF***	ABC transport system, ATP-binding protein	22.14
**Bbr_0168_** ***nanA***	*N*-acetylneuraminate lyase	15.17
**Bbr_0169_** ***nagB1***	Glucosamine-6-phosphate isomerase	17.77
**Bbr_0171_** ***nanH***	Sialidase	7.23
**Bbr_0173_** ***nanR***	Transcriptional regulator, GntR family	4.80
**Bbr_1247_** ***nagA2***	*N*-acetylglucosamine-6-phosphate deacetylase	4.59
**Bbr_1248_** ***nagB3***	Glucosamine-6-phosphate isomerase	6.08
**Bbr_1585_** ***galE***	UDP-Glc-4-epimerase	3.96
**Bbr_1586_** ***nahK***	*N*-acetylhexosamine kinase	3.67
**Bbr_1587_** ***lnbP***	Lacto-*N*-biose phosphorylase	3.19
**Bbr_1588**	ABC transport system, permease protein	3.34
**Bbr_1589**	ABC transport system, permease protein	2.88
**Bbr_1590**	ABC transport system, solute binding protein	8.95
**Bbr_1740_** ***dapA4***	Dihydrodipicolinate synthase	9.91
**Bbr_1741**	Conserved hypothetical protein	8.83
**Bbr_1742_** ***fucP***	L-fucose permease	7.81
**Bbr_1833_** ***lacZ7***	Beta-galactosidase	4.31
**Bbr_1879**	PTS system, glucose-specific IIABC component	6.97
**Bbr_1880**	PTS system, GlcNAc-specific IIBC component	18.15
**Bbr_1884_** ***galT2***	Gal-1-phosphate uridylyltransferase	3.86

The cut-off point for the level of up-regulation is 2.5-fold with a *P*-value of <0.001.

### Analysis of the monosaccharide composition of fermented and non-fermented mucin

**Figure 2:**
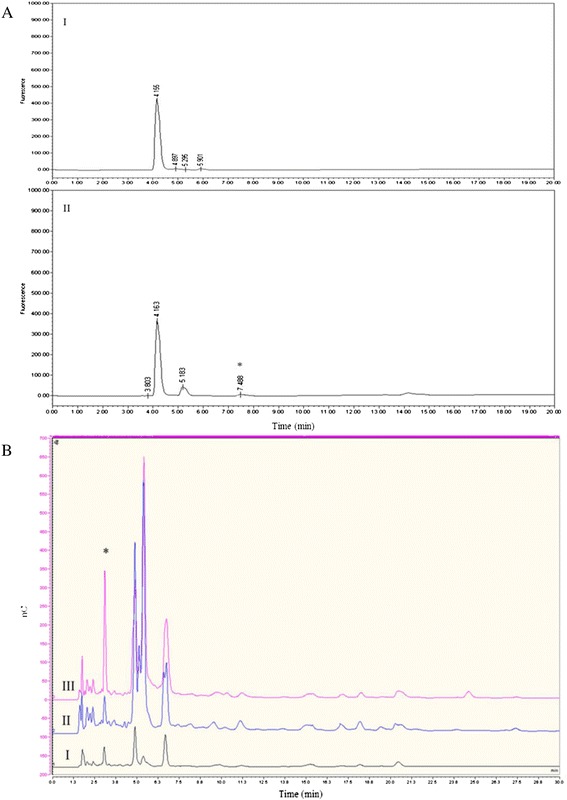
**Analysis of the monosaccharide composition of fermented and non-fermented mucin. (A)** HPLC profile of 2-AB -labelled (I) mMRS supplemented with 0.4% mucin and (II) the media from (I) after 30 h growth of *B. bifidum* PRL2010-pPKCM7. The peak for Fuc is marked with *. **(B)** Qualitative HPAEC-PAD analysis of (I) mMRS supplemented with 0.4% mucin, (II) the media from (I) following 30 h growth of *B. bifidum* PRL2010-pPKCM7 and *B. breve* UCC2003-pAM5 in co-culture, (III) the media from (I) after 30 h growth of *B. bifidum* PRL2010-pPCM7. The peak for Fuc is marked with *. nC, nanocoulombs.

HPAEC-PAD was used to quantitatively analyze the carbohydrate profile of non-fermented mucin, compared to the cell free supernatant (CFS) of *B. bifidum* PRL2010-pPKCM7 grown in mucin for 30 h. Non-fermented mucin was shown to contain 0.13 nmol mg^−1^ of free Gal and 3.96 nmol mg^−1^ of free Glc, both of which support growth of *B. breve* UCC2003 [51], thus presenting a plausible explanation for the initial increase in *B. breve* UCC2003-pAM5 cell numbers during the first 12 h of incubation in mucin-containing medium (Table 4). However, after 30 h growth of *B. bifidum* PRL2010-pPKCM7, the carbohydrate profile of the medium was significantly altered. Glc was no longer detected, indicating its uptake by *B. bifidum* PRL2010-pPKCM7, but the amount of free Gal increased to 51.73 nmol mg^−1^, while 34.94 nmol mg^−1^ of free Fuc was also detected, indicative of the extracellular glycosidase activity of *B. bifidum* PRL2010-pPKCM7 (Table 4). A consistent pattern was observed in regards to the amount of Fuc and Gal released by *B. bifidum* PRL2010 activity. The presence of Fuc was further verified by fluorescent labelling of the CFS samples and analysis by RP-HPLC (Figure 2A). It was also confirmed that free Fuc was absent in non-fermented mMRS supplemented with 0.4% mucin, which indicated that its presence was a result of *B. bifidum* PRL2010-pPKCM7 extracellular activity (Figure 2A). To investigate whether these released monosaccharides from mucin support the observed growth and viability of *B. breve* UCC2003-pAM5 in co-culture, the carbohydrate profile of the CFS of the co-culture was compared to that of *B. bifidum* PRL2010-pPKCM7 grown independently in mucin (hence any difference observed could be attributed to the presence of *B. breve* UCC2003-pAM5). The results support the hypothesis that *B. breve* UCC2003-pAM5 is cross-feeding on at least one carbohydrate released following degradation of mucin by *B. bifidum* PRL2010-pPKCM7. After 30 h Fuc was present in the carbohydrate profile of *B. bifidum* PRL2010-pPKCM7, but absent (or at least below the detection level) in that of the co-culture, indicative of its uptake and utilization by *B. breve* UCC2003-pAM5 (Figure 2B).

**Table 4:** **Quantification of Fuc, Gal and Glc by HPAEC-PAD**

	**Fuc**	**Gal**	**Glc**
**mMRS +0.4% mucin (non-fermented)**	n.d.	0.13	3.96
**mMRS +0.4% mucin (30 h growth of** ***B. bifidum*** **PRL2010)**	34.94	51.73	n.d.

Concentrations given in nmol mg^−1^. n.d. = not detected.

### Growth of *B. breve* UCC2003 mutants in co-culture in mucin with *B. bifidum* PRL2010-pPKCM7

**Figure 3:**
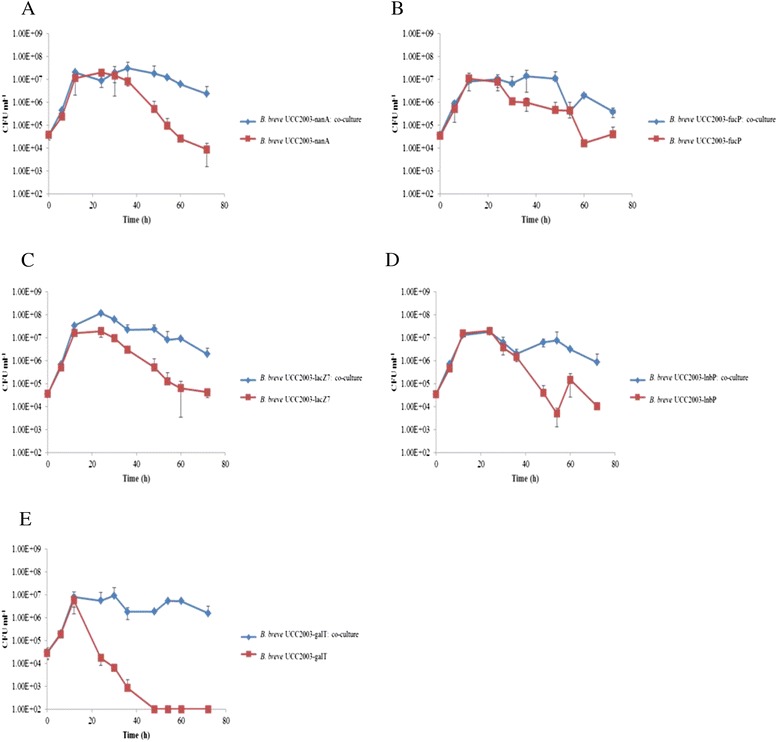
**Individual and co-culture growth profiles of** ***B. breve*** **UCC2003 mutant strains in mucin.** Growth profiles of the mutants **(A)** *B. breve* UCC2003-nanA, **(B)** *B. breve* UCC2003-fucP, **(C)** *B. breve* UCC2003-lacZ7, **(D)** *B. breve* UCC2003-lnbP and **(E)** *B. breve* UCC2003-galT in mMRS supplemented with 0.4% mucin, separately or in co-culture with *B. bifidum* PRL2010-pPKCM7, over 72 h. The results presented are the mean values of duplicate experiments. Error bars represent standard deviation.

**Figure 4:**
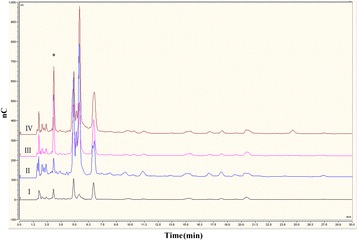
**Analysis of the monosaccharide composition of fermented and non-fermented mucin including** ***B. breve*** **UCC2003-fucP.** Qualitative HPAEC-PAD analysis of (I) mMRS supplemented with 0.4% mucin, (II) the media from (I) following 36 h growth of *B. breve* UCC2003-pAM5 and *B. bifidum* PRL2010-pPKCM7 in co-culture, (III) the media from (I) following 36 h growth of *B. bifidum* PRL2010-pPKCM7, (IV) the media from (I) following 36 h growth of *B. breve* UCC2003-fucP and *B. bifidum* PRL2010-pPKCM7 in co-culture. The peak for Fuc is marked with *. nC, nanocoulombs.

HPAEC-PAD and transcriptome data suggested that the improved growth and viability of *B. breve* UCC2003-pAM5 in co-culture was a result of the strain cross-feeding on Fuc, while microarray data suggested sialic acid, Gal, GNB and/or another of the Gal-containing constituents of mucin may also be utilized. To determine which, if any, of these sugars was primarily responsible for growth of the strain in co-culture, insertion mutants were constructed in the *fucP, lnbP* and *lacZ7* genes, resulting in strains *B. breve* UCC2003-fucP, *B. breve* UCC2003-lnbP and *B. breve* UCC2003-lacZ7. Two additional mutants, *B. breve* UCC2003-galT, which harbours a transposon in a predicted Gal-1-phosphate uridyltransferase-encoding gene, designated *galT1* and was shown to be incapable of growth in Gal [41], and the previously described insertion mutant, *B. breve* UCC2003-nanA, which cannot utilize sialic acid [13], were also tested. The insertion mutants were individually analyzed for their ability to grow in mucin in the presence or absence of *B. bifidum* PRL2010-pPKCM7. In the absence of *B. bifidum* PRL2010-pPKCM7, the mutant strains behaved similarly to *B. breve* UCC2003-pAM5, as initial growth over the first 12 h was followed by a decline in viable cells to 10^4^ CFU ml^−1^ or lower at 72 h (Figure 3). It was also found that removing the ability of the strain to utilize a particular sugar did not significantly affect its ability to grow in co-culture with *B. bifidum* PRL2010-pPKCM7. Similar to *B. breve* UCC2003-pAM5, all mutant strains attained a viable count of between 10^7^ and 10^8^ CFU ml^−1^ between 12 h and 30 h, and maintained a viable count of 10^6^ CFU ml^−1^ until 72 h, with the exception of *B. breve* UCC2003-fucP which dropped to 10^5^ CFU ml^−1^ (Figure 3). The carbohydrate profiles of each mutant grown in co-culture were also analyzed by HPAEC-PAD and it was found that each mutant, with the exception of *B. breve* UCC2003-fucP, produced an identical carbohydrate profile to that produced by the *B. breve* UCC2003-pAM5. *B. breve* UCC2003-fucP, as expected, was not capable of utilizing/transporting Fuc, as evident from the presence of Fuc in its carbohydrate profile (Figure 4).

## Discussion

Early investigations into the degradation of mucin established the role of extracellular glycosidases produced by a sub-population of bacteria in the gut, suggesting that the remaining enteric bacteria may cross feed on the released oligosaccharides [19,22]. Since then, much of the research into bacterial degradation of mucin has focused on the characterization of such extracellular glycosidases, such as two α-L-fucosidases and an exo-α-sialidase from *B. bifidum* JCM1254, and an endo-α-*N-*acetylgalactosaminidase from *B. longum* JCM1217 [23,24,61,62]. Another study identified, by a genomic as well as a transcriptomic and proteomic approach, a number of extracellular enzymes involved in mucin degradation by *B. bifidum* PRL2010, including a predicted cell wall-anchored endo-α-*N*-acetylgalactosamidase, two α-L-fucosidases, two exo-α-sialidases, a β-galactosidase and two putative *N-*acetyl-β-hexosaminidases, all of which contained a signal peptide [26].

Extracellular enzymes similar to those outlined above were not found in the genome of *B. breve* UCC2003 [33]. Therefore, it was not surprising that *B. breve* UCC2003-pAM5 was incapable of high density growth in a medium containing mucin as the sole carbon source. However, when *B. bifidum* PRL2010-pPKCM7 was included as a co-cultivating, mucin-degrading bacterium, the growth and viability of *B. breve* UCC2003-pAM5 was improved compared to the control situation. A combination of HPAEC-PAD and transcriptome analyses identified some of the possible monosaccharides and oligosaccharides which could support this enhanced co-cultivation growth/viability phenotype of *B. breve* UCC2003-pAM5, represented by sialic acid, Fuc, Gal and/or Gal-containing constituents of mucin.

HPAEC-PAD analysis identified two monosaccharides, namely Fuc and Gal, which were released from mucin by *B. bifidum* PRL2010-pPKCM7 activity. Fuc was shown to be internalized by *B. breve* UCC2003-pAM5 during growth in co-culture, suggesting a role in the enhanced growth/viability phenotype of *B. breve* UCC2003-pAM5 in co-culture. Transcriptome and mutagenesis data supports this hypothesis, as shown by increased transcription of a cluster (Bbr_1740-1742) involved in the uptake and utilization of Fuc and by the inability of a mutant in the *fucP* gene to internalize Fuc from the growth medium.

Gal has previously been shown to support the growth of *B. breve* UCC2003 [51]. However, given that Gal is ubiquitous in mucin, it may be internalized by *B. breve* UCC2003-pAM5 as a monosaccharide and/or as a constituent of a larger oligosaccharide. Interestingly, transcriptome data revealed increased transcription of the Bbr_1585-1590 gene cluster, which includes predicted GalE, NahK and LnbP-encoding genes, all of which are required for the metabolism of GNB [57], as well as an adjacent predicted ABC transport system. Outside of this cluster, the predicted *galT2* gene (Bbr_1884) was also up-regulated. A previous study in *B. bifidum* hypothesized that GalT2 is specifically required for the metabolism of GNB from mucin [63]. Up-regulation of each of the required genes for the complete GNB pathway, as well as a predicted transport system, suggests that GNB might be the preferred source of Gal for *B. breve* UCC2003 in co-culture. A predicted β-galactosidase-encoding gene, *lacZ7,* was also up-regulated in co-culture, implying that *B. breve* UCC2003 may utilize another Gal-containing oligosaccharide. However, the precise substrate or preferred linkage of this enzyme is still a matter of speculation. Gal-containing oligosaccharides and GNB were not identified in the HPAEC-PAD data. However, the presence of four predicted β-galactosidases on the genome of *B. bifidum* PRL2010, as well as a clear homolog of the GNB pathway [26], suggests that *B. bifidum* PRL2010 also utilizes these sugars, and it is therefore possible that in co-culture the two strains compete for such sugars.

Interestingly, transcriptome data also revealed increased transcription of two clusters previously shown to be up-regulated in the presence of sialic acid [13]. This is inconsistent with the HPAEC-PAD data, in which sialic acid was not identified; however, it should be noted that the mucin from porcine stomach used in this study contains only 0.5%- 1.5% bound sialic acid, and it is thus possible that the released sialic acid is below the detection level of the HPAEC-PAD system. However, since *B. bifidum* PRL2010 has been shown to be incapable of using sialic acid [13,26], it seems likely that *B. breve* UCC2003 would utilize this sugar, even if available in very low quantities.

While accepting the HPAEC-PAD and transcriptome data are not definitive, the results suggest that *B. breve* UCC2003-pAM5 utilizes a combination of sugars in co-culture, a suggestion supported by the fact that none of the mutants tested displayed a different phenotype to *B. breve* UCC2003-pAM5 in co-culture. Construction of double or triple mutant strains is expected to result in strains that are impaired in growth/viability under co-culture conditions, however, at present such multiple mutant construction is not technically feasible for *B. breve* UCC2003 (and have to the best of our knowledge not been described for any bifidobacterial species/strain).

Our results highlight the different approaches taken by two species of bifidobacteria to proliferate and survive in the gut. *B. bifidum* PRL2010 has been shown to utilize mucin oligosaccharides (and structurally similar HMOs), a highly complex, yet ubiquitous carbohydrate source in the gut. Aside from host glycans such as mucin and HMO, however, the fermentation ability of *B. bifidum* PRL2010 is rather limited [26,52]. *B. breve* UCC2003, on the other hand, is a more versatile bacterium from a metabolic perspective, capable of utilizing a number of host and diet-derived carbohydrates. As seen in this study it can scavenge constituents of mucin released by the extracellular glycosidase activity of other bifidobacteria, as well as cross-feed on certain HMO [13], a characteristic which reflects the abundance of representatives of this species in the infant gut [5]. However, *B. breve* UCC2003 also has the ability to utilize a number of plant-derived carbohydrates such as starch, galactan, cellodextrins and raffinose [7,8,10,11]. *B. breve* strains have been identified in the adult human bifidobacterial population, although the relative abundance is lower than in infants [5,64]. The similar phenotype of *B. breve* UCC2003-pAM5 and the mutant strains in co-culture also highlights the adaptability of the strain, emphasizing its ability to switch to a different carbon source, depending on the carbohydrates available. This versatility is reflected on the genome of *B. breve* UCC2003, which contains a large number of carbohydrate utilization clusters [33], suggesting an ability to alternate between and/or co-utilize diet- and host- derived carbohydrates depending on availability. The gut commensal *Bacteroides thetaiotaomicron*, displays a remarkable metabolic flexibility, whereby in the absence of a dietary-derived fibre, this bacterium will shift its metabolic activities towards the degradation of mucin [65]. These results suggest that *B. breve* UCC2003 may also be capable of such flexibility, although further study is required.

The ability to degrade mucin seems to be limited to particular gut commensals, such as certain species of *Bacteroides, Bifidobacterium, Ruminococcus* and *Akkermansia* [20,26,66-68]. Pathogenic bacteria appear to be poorly adapted to mucin degradation [69], however, multiple studies have shown pathogens utilizing constituents of mucin released by commensal glycosidases. For example, *C. jejuni* has been shown to utilize Fuc as a substrate for growth [55], while enterohaemorrhagic *E. coli* uses Fuc as a signal to induce virulence [70]. Similarly, *Salmonella typhimurium* and *Clostridium difficile* have been shown to utilize sialic acid released by the sialidase activity of *Bacteroides thetaotaomicron* in a gnotobiotic mouse [71]. The uptake of mucin-derived Fuc by *B. breve* UCC2003, as well as the presumable utilization of mucin-derived sialic acid, suggests a potential role for *B. breve* UCC2003 in “mopping up” the released constituents of mucin, providing competition to potential pathogens and inhibiting or limiting their proliferation, although further study is required to expand on this hypothesis.

The mutually beneficial relationship between the host and the intestinal bacteria has been well established [72], however, it is of equal importance to acknowledge the symbiotic relationships formed between genera and species of the intestinal bacteria. Cross-feeding between *B. breve* UCC2003 and *B. bifidum* PRL2010 has now been shown for mucin in the present work and previously for 3′ sialyllactose [13], and given the structural similarity between mucin oligosaccharides and HMOs, it may be assumed that *B. breve* UCC2003 has the ability to cross feed on other HMO-derived constituents that are released by *B. bifidum* PRL2010. These results highlight the compatibility of these two species of bifidobacteria, especially when viewed in contrast to the strategy employed by *B. longum* subsp. *infantis*, which assimilates HMOs in their intact form, leaving none for potential cross-feeding with another species [73]. Interestingly, another type of cross-feeding has been described, whereby *Eubacterium halii* utilizes lactate produced by *Bifidobacterium adolescentis* during growth on starch, resulting in butyrate production by *Eu. halii* [74]. Butyrate is a major source of energy for colonocytes and has also been implicated in protection against colonic carcinogenesis [75,76]. Understanding such complex interactions between different members of the intestinal bacteria is of crucial importance when attempting to influence the activity or composition of the intestinal microbiota, such as in the use of probiotics.

## Conclusion

This study provides *in vitro* proof for the existence of a commensal relationship between two species of bifidobacteria in the large intestine, namely *B. breve* and *B. bifidum*, in which *B. breve* UCC2003 benefits from the carbohydrates released by the extracellular glycosidase activities of *B. bifidum* PRL2010. To our knowledge, this is the first study to describe the molecular details of such cross-feeding, with particular emphasis on the carbohydrate components which support the improved growth and survival in co-culture. The results shown here improve our knowledge on how *B. breve* UCC2003 colonizes the (infant) gut in the absence of dietary-derived carbohydrates, and also emphasize this strain’s ability to switch between carbohydrate sources depending on availability. This is an advantageous characteristic in terms of enhanced survival and colonization ability in both the infant and adult gut, and may present an advantage to the host in limiting opportunities for pathogenic microbes to proliferate in the gut.
